# A Critical Review on the Production of Electrospun Nanofibres for Guided Bone Regeneration in Oral Surgery

**DOI:** 10.3390/nano10010016

**Published:** 2019-12-19

**Authors:** Federico Berton, Davide Porrelli, Roberto Di Lenarda, Gianluca Turco

**Affiliations:** Clinical Department of Medical, Surgical and Health Sciences, University of Trieste, 34100 Trieste, Italy; dporrelli@units.it (D.P.); rdilenarda@units.it (R.D.L.); gturco@units.it (G.T.)

**Keywords:** electrospinning, guided bone regeneration, oral surgery, membranes, scaffolds

## Abstract

Nanofibre-based membranes or scaffolds exhibit high surface-to-volume ratio, which allows an improved cell adhesion, representing an attractive subgroup of biomaterials due to their unique properties. Among several techniques of nanofiber production, electrospinning is a cost-effective technique that has been, to date, attractive for several medical applications. Among these, guided bone regeneration is a surgical procedure in which bone regeneration, due to bone atrophy following tooth loss, is “guided” by an occlusive barrier. The membrane should protect the initial blood clot from any compression, shielding the bone matrix during maturation from infiltration of soft tissues cells. This review will focus its attention on the application of electrospinning (ELS) in oral surgery bone regeneration. Despite the abundance of published papers related to the electrospinning technique applied in the field of bone regeneration of the jaws, to the authors’ knowledge, no articles report clinical application of these structures. Moreover, only a few records can be found with in vivo application. Therefore, no human studies have to date been detectable. New approaches such as multifunctional multilayering and coupling with bone promoting factors or antimicrobial agents, makes this technology very attractive. However, greater efforts should be made by researchers and companies to turn these results into clinical practice.

## 1. Introduction

Nanofibres in tissue Eegineering (TE) represent an extremely attractive subgroup of biomaterials due to their unique intrinsic features. Nanofibre-based membranes or scaffolds exhibit high surface-to-volume ratio, which allows an improved cell adhesion. Moreover, these structures can be implemented with proteins, drugs and ligands. The mechanical and morphological properties of these structures are even more promising thanks to the customizable dimensions, orientation, packing, porosity and density of the fibres. Finally, the resulting three-dimensional structure of the obtained nanostructured material mimics the morphology of the extracellular matrix, which consists predominantly of collagen fibrils, coupled with elastin and other macromolecules such as glycoproteins [1]. Furthermore, nanofibres can promote specific cellular functions such as adhesion, proliferation, differentiation, and can modulate stem cell behavior [2,3].

Several techniques have been proposed in literature to fabricate nanofibres: phase separation technique [4], self-assembly fibres [5], template synthesis [6] and electrospinning (ELS) [7] to name some. Among these techniques, electrospinning is a cost-effective technique that can be used to prepare nanofibres. The ELS technique has risen its popularity since its early development in the 1930s [8] along with the refinements of its basic components and setup.

This technique is used for polymeric solutions that can be modified and enriched with bioactive molecules. Electrospun fibres are to date, attractive for several medical applications such as, wound dressings, drug delivery and scaffolds for tissue engineering [9]. Thanks to their features, electrospun nanofibres have been attractive also in the dental field: periodontal regeneration [10], coatings for caries prevention [11], enrichment of resin composites [12], implant surface modification [13], wound healing of mucosa [14], drug-releasing systems [15] and bone regeneration [16] are the main topic of basic research. Guided bone regeneration (GBR) is a surgical procedure in which bone regeneration, due to bone atrophy following tooth loss, is “guided” by an occlusive barrier. The membrane should maintain the shape of the defect in which the bone is stimulated to regenerate. The membrane should also protect the initial blood clot from any compression, shielding the bone matrix during maturation from infiltration of soft tissues cells. Therefore, these membranes should maintain suitable mechanical properties at least for three months of permanence exhibiting at the same time a proper bio-degradability which avoids second surgery for patients [17,18]. This review will focus its attention on the production of ELS membranes for bone regeneration in oral surgery of ELS in oral surgery bone regeneration. Hereafter, the production of both scaffolds and membranes by means of ELS is discussed in view of regenerating alveolar bone defects. [19,20], prior to implant insertion in the atrophic jaws [21].

## 2. Principles of the Electrospinning (ELS) Technique

**Figure 1:**
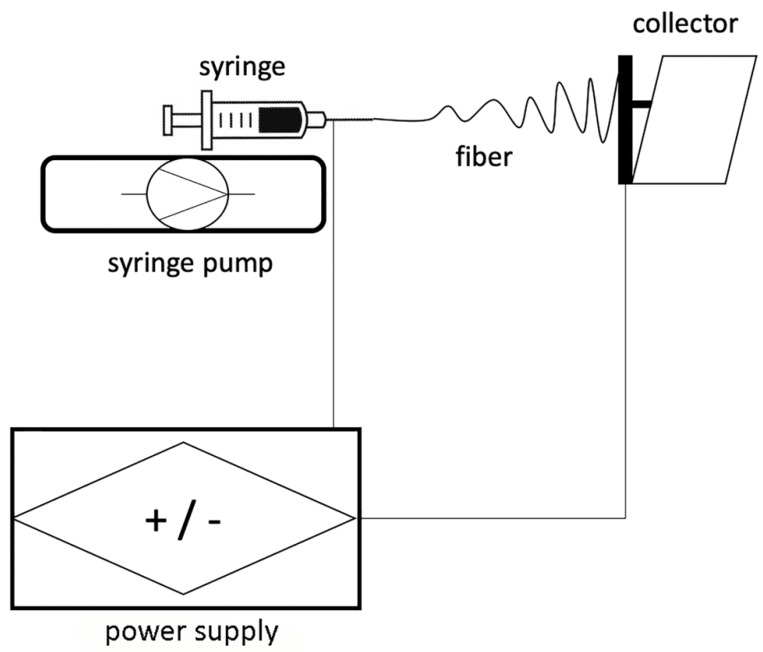
Schematic representation of the essential set-up of an electrospinning (ELS) device.

This technique was firstly applied in 1934 by Anton Formhals and represents a combination of two techniques which are the electrospray and the spinning of fibres [22]. A high electric field is applied to both the syringe needle, which contains a polymeric solution, and to the collector (Figure 1).

**Figure 2:**
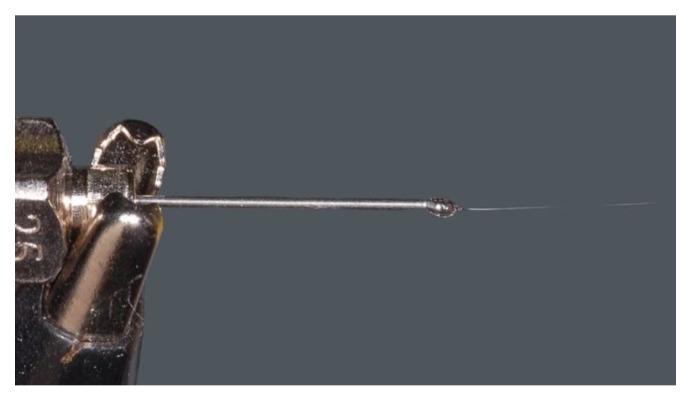
Taylor cone obtained with the following parameters: a solution of polycaprolactone (PCL) 12% *w*/*v* in dichloromethane/dimethylformamide (DCM/DMF) 7:3 applying 17 kV of potential and 0.6 mL/h of flow rate and using a 25 G needle. Nikon D3500, macro 105 Sigma tamron lens, Sigma ring flash.

The collector and the syringe needle are kept at the proper distance one from the other. Metallic plates, aluminum foils and rotating drums can be used as target for the collection of nanofibres during the electrospinning process. The potential difference is, hence, able to overcome the surface tension electrostatic forces of the polymeric solution ejected from the needle tip and assume the so called “Taylor cone” configuration [23] (Figure 2). This process shapes the polymeric solution into a jet of charged fluid that is electrostatically attracted by the collector. The solvent evaporates during this transit from the needle to the collector allowing for the accumulation of dry fibres on it.

## 3. Variables Influencing the Electrospinning Technique

Despite the broad spectrum of polymers that can be electrospun, an equilibrium between the physical and chemical properties and the ratio between the solute and the solvents has to be thoroughly sought, along with the multiple variables that may affect the final morphology of the fibres obtained. A list of the relevant parameters is provided in the following table (Table 1) [24,25,26].

The final goal of the process is the fabrication of nanofibres with diameters at the nanoscale and without the presence of defects (e.g., beads, which are the expression of incomplete solvent evaporation). Precise choice of the principal polymer and its adequate solvents should be settled in order to obtain limited surface tension, adequate viscosity and charge density. This has to favour the formation of a continuous flow, which must not collapse in droplets, or beads, after potential difference administration. Both viscosity and surface tension, in conjunction with polymer molecular weight, polymer concentration, conductivity of the solution, influence the fibre morphology and porosity.

Molecular weight depends on the chain length of the polymer and can be related to the entanglements of the molecules. This fact explains why high molecular weight results in viscous solutions compared to low molecular weight. Therefore, the molecular weight of the polymer should be correctly considered for the selection of solvents and concentrations. Indeed, if the solution exhibits too high a viscosity, this will hamper the flow through the capillary and the polymer may dry up or drip at the needle tip. Conversely, solutions with relatively low concentration will result into droplets.

**Figure 3:**
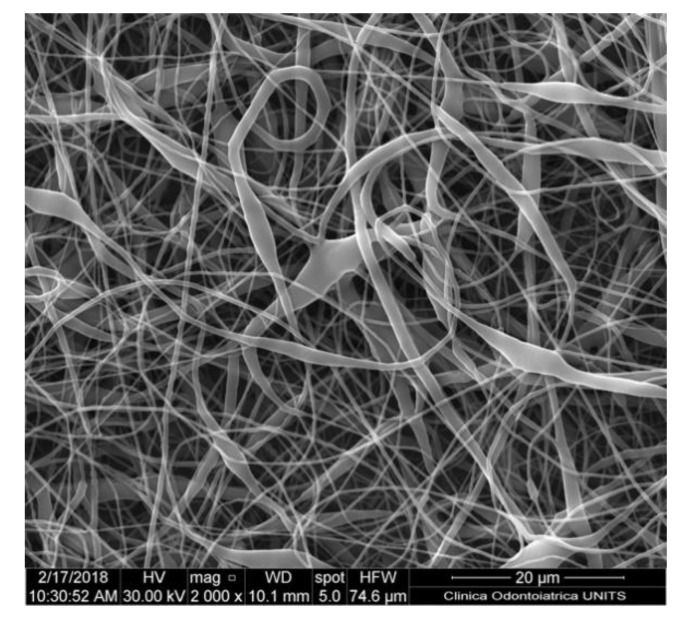
Nanofiber-based membrane obtained with the following parameters: chitosan 2.5% *w*/*v* + lactose-modified chitosan 0.5% *w*/*v* in acetic acid 90%, 15 kV of potential, 27 G needle, 0.6 mL/h of flow rate. Ribbon-like fibres can be appreciated. Quanta250 scanning electron microscope (SEM), FEI, Hillsboro, OR, USA; 2000×.

**Figure 4:**
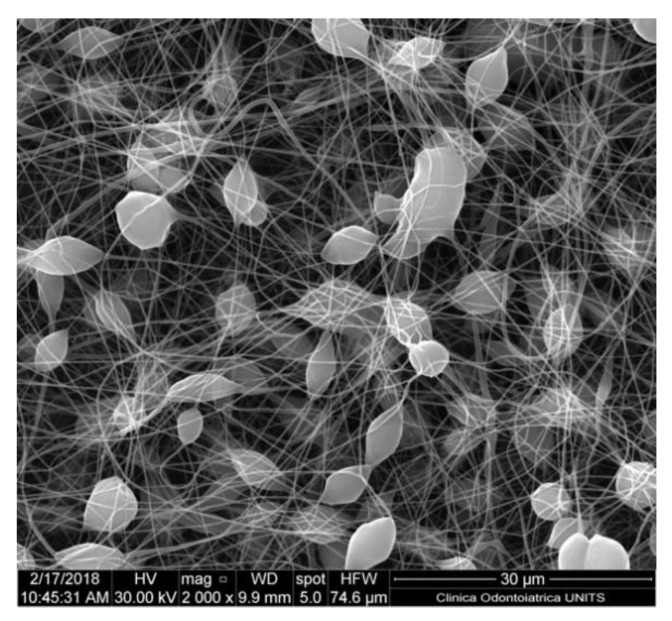
Nanofiber-based membrane obtained with the following parameters: PCL 6% *w*/*v* in DCM/methanol (MeOH) 7:3, 17 kV of potential, 27 G needle, flow rate of 0.6 mL/h. The formation of multiple beads can be appreciated. Quanta250 SEM, FEI, Hillsboro OR, USA; 2000×.

Solubility and boiling point of the solvent are paramount factors. Volatile solvents are ideal options due to rapid evaporation during the transit from the needle tip to the collector [27]. High boiling points solvents may not evaporate completely prior to hitting the target, resulting in flat ribbon shape (Figure 3) fibres instead of circular fibres, the presence of beads or other defects (Figure 4) [28]. The volatility of the solvent may affect the final microscopic characteristics of the obtained fibres including porosity, shape and size.

Besides the aforementioned process parameters, which are mainly related to the polymeric solution, several others have to be taken into consideration: voltage, distance between the needle tip and the collector, flow rate, needle gauge and type of collector. Starting from the latest, in the conventional ELS set up, collectors can be static, round shaped and covered by a common aluminum foil. According to the final macroscopic structure of the biomaterial, however, the collector can be oscillating, or rotative and can be flat or cylindrical. Nanowire-in-microtubes can be obtained in contrast with co-electrospinning: an alternative setup which allows for the production of core-shell fibres and inner hollow fibres [29,30,31]. The electrodes applied both on the metallic needle and on the collector, bring the potential difference from the energy supply. Many combinations of voltage can be administered: usually from 7 kV to 35 kV, distributed equally or diversely between the needle tip and the collector. The flow rate of the syringe pump should be regulated in accordance with the applied voltage in order to maintain a continuous collection of fibres on the target. Comparing low with high voltages, these latter result in a smaller Taylor cone and, therefore, in thinner fibres with higher rate of deposition on the target. However, critical voltage may vary among polymeric solutions. The formation of thinner nanofibers with an augmentation of the voltage applied is attributed to the stretching of the polymer solution in correlation with the charge repulsion within the polymer jet. The increases in the diameter and formation of beads with an increase in the applied voltage are attributed to the decrease in the size of the Taylor cone and increase in the jet speed for the same flow rate [32]. The higher the voltage, the larger the fibre diameter due to the increased feed rate. Conversely, the increment of the flow rate would turn in occurrence of defects (beads) because of the improper evaporation of the solvent, prior to the fibre deposition [25]. The proper regulation of the flow rate is also function of the distance between the metallic needle and the collector. This parameter should allow for the correct solvent evaporation during the transit between the source and the target.

Depending on the polymer and the solvent, the needle diameter can vary. Smaller internal diameter reduces the probability of occlusion of the spinneret due to less exposure time of the jet to the environment. Reduction in needle internal diameter increases the surface tension of the solution corresponding to a smaller droplet. This causes the acceleration of the jet to decrease. Therefore, the jet gains more flight time before deposition; this results in smaller diameter of the fibres [26]. Usual needle diameters are reported to be from 18 G to 30 G [33,34,35].

In order to stabilize the Taylor cone, the flow rate needs to be adjusted in a correct range. A constant and stable flow rate is necessary to minimize the beads formation in the electrospun web of fibres [25]. A lower flow rate is preferable to let the solvent evaporate properly [36]. However, there should always be a minimum feed rate of the spinning solution. It has been observed that the fibre diameter and the pore diameter (i.e., the void portion of the structure) increase with increased polymer flow rate and by changing it, the morphological structure can be slightly altered. Few studies have systematically investigated the relationship between solution feed or flow rate and the fibre morphology and size [37,38]. Nonetheless, it can be stated that high flow rates result in beads due to the not optimal drying time prior to fibre accumulation on the target [39,40]. Along with the feed rate, the needle-to-collector distance also affects the solvent’s evaporation: as the distance increases, using the same voltage the magnitude of the electric field decreases. However, the effect of needle-to-collector distance on fibre morphology is not as significant as the other parameters. Common reported distances for solution ELS are 15 cm to 30 cm [33]. Another important variable is represented by conductivity of the solution. High conductivity enables polymer solutions to carry greater charge compared to low conductivity. Therefore, high conductivity yields greater tensile forces and a reduction in nanofibre diameter [41]. Generally, electrospun nanofibres with the smallest fibre diameter can be obtained with the highest electrical conductivity and it has been found that the jet radius varies inversely with the cube root of the electrical conductivity of the solution [42]. Conductivity of polymer solution can also be enhanced using surfactants. The approach of increasing the solution conductivity by salt addition has also been explored for polymers such as, polyoxyethylene oxide (PEO) [43], collagen type I-PEO [44], PVA [45], polyacrylic acid (PAA) [46], polyamide-6 [47] and others.

Apart from solution and processing parameters, also the ambient parameters (e.g., humidity, pressure and temperature) influence the fibre morphology. The variation in humidity while spinning polystyrene solutions was studied and it was showed that an increase in the humidity results in the appearance of small circular pores on the surface of the fibres; a further increase in the humidity leads to pore coalescence [48]. In 2004, the effect of temperature, ranging from 25 to 60 °C, was investigated on the ELS of polyamide-6 fibres and it was found that higher temperatures led to smaller fibre diameter. The authors attributed this phenomenon to the decrease in the viscosity of the polymer solutions at increased temperatures [47].

## 4. Properties of Electrospun Materials

The characterization of fibres produced by the electrospinning process remains one of the most difficult tasks, as the chances of harvesting single fibres are rare. Generally in electrospinning, the polymers used are characterized in three terms: physical and structural, mechanical, and chemical [49]. Moreover, biological properties of the biomaterial obtained should be determined before in vitro and in vivo research. As mentioned above, electrospun nanofibres show remarkable micro and nano structural characteristics, high surface area, small pores size, and the possibility of producing three-dimensional structures that enable the development of advanced materials for sophisticated applications. One of the most apparent advantages of electrospun scaffolds is the ability to mimic extra-cellular matrix (ECM). Pores distribution, size and fibre orientation independently affect adhesion, proliferation and differentiation of cells. Hydrophilicity of the final biomaterial facilitates cellular adhesion; by contrast, hydrophobic surface may hamper cellular or bacterial colonization; this effect could be desired in certain circumstances (e.g., outer layer of membranes in guided bone regeneration). Nonetheless, surface hydrophilicity may be easily augmented with fast and easy treatments such as air plasma treatment [50,51]. Fibre orientation plays also an important role on cellular behavior. It has been shown that, although osteoblast proliferation is comparable on aligned and randomly distributed fibres, a higher calcium production has been detected when the cells are seeded on aligned fibres [52].

Electrospun nanofibres can be fabricated in a wide range of diameters from micro to nano-scale, varying electrospinning process parameters and polymer solutions. The microscopic characteristics of nanofibre are highly dependent on fibre morphology, diameter and surface area [53]. Indeed, nanofibre diameter is inversely proportional to surface area and it is a meaningful indicator of the degradation of electrospun biomaterials. For the characterization of geometric morphology, techniques such as scanning electron microscopy (SEM), transmission electron microscopy (TEM), and atomic force microscopy (AFM) are commonly used [14,38,54]. For SEM, samples have to be electrically conductive, therefore, for most of the electrospun polymers, a gold or platinum coating must be applied. Moreover, SEM is a quick method for observing the fibres produced and it requires a very small sample size for its analysis.

Fourier transform infrared (FTIR) and nuclear magnetic resonance (NMR) techniques can be used for the analysis of the nanofibre molecular structure [55]. These methods are able to detect not only the single structures but also the inter-molecular interactions. Given the fact that in the biological environment degradable electrospun fibres are chemically degraded by enzymes such as lysozyme, biocompatibility of the products is crucial [56]. The chemical properties of electrospun fibres are mainly influenced by two factors: hydrophilicity and chemical composition of the fibres. Above all, a controlled degradation process is one of the most important goals in bone regeneration, as far as for tissue regeneration, in which the scaffold should be subsequently substituted by the newly formed tissue. Therefore, the bio-degradability of scaffolds brings advantages in terms of decreased morbidity for the patient, time and costs. In fact, non-adsorbable scaffolds have to be removed with a second surgery that implies more stress for the patient, together with the increase of the whole cost of the final surgical treatment.

The characterization of the mechanical features is critical for the electrospun nanofibres conceived for bone regeneration. In fact, these implantable devices have to act as space-making and space-keeping material (against gravity, competitor tissues growth and muscle activity), and to maintain a separation between different cell and tissue types. Hence, mechanical properties should be optimized and characterized. Mechanical characterization is usually performed by tensile tests, using specimens prepared from the electrospun nanofibres, with certain attention of the sample manipulation. Other approaches have been validated for the mechanical characterization of nanofibres by employing nanoindentation, bending tests, resonance frequency measurements, and microscale tension tests. Many authors reported that there is no anisotropy in the in-plane tensile behavior when the nanofibrous membranes are collected on a static collector, simplifying the Young’s modulus evaluation. In order to improve the mechanical and handling properties of electrospun nanofibres, different strategies can be applied. Cross-linking agents, such as carbodiimide or aldehydes, can be used to increase the tensile and flexural strengths of fibres [57]. Furthermore, electrospun gelatin scaffolds cross-linked with genipin display enhanced mechanical properties preserving their morphology after being soaked in water, in respect to the non-cross-linked ones [58].

Finally, biocompatibility and biological properties are essential for these biomaterials. As stated before, nanofibres mimic ECM structure, but their chemical and morphological characteristics affect cellular response. Hence, cell attachment and proliferation represent one of the biological final goal of these biomaterials. A major advantage of using nanofibres is linked to their high surface-to-volume ratio which allows the extensive absorption of proteins (such as bone morphogenetic proteins, BMPs) and promote the formation of cellular binding sites. For membranes conceived to act as a cellular barrier, porosity is extremely important. Indeed, these structures should guarantee isolation of fibroblasts belonging to the soft tissue that may colonize the bone defect and hamper bone regeneration. Hence, membranes for guided bone regeneration need to be permeable for fluids and molecules but not for fast-turnover cells.

## 5. Electrospinning in Guided Bone Regeneration (GBR)

Despite the abundance of published papers related to the electrospinning technique applied in the field of bone regeneration of the jaws, at the date of writing this manuscript and to the authors’ best knowledge, no articles report clinical application of these structures. Moreover, only a few records can be found with in vivo application [16,59,60,61,62,63]. Therefore, no human studies have to date been detectable. Hence, the risen question is why more than a decade of published papers on ELS in bone regeneration field has not turned in clinical trials yet? The main reason, which will better discussed below, lies in the difficulties related to the approval of these products. A narrative review published in 2016 [26] reported only 8 papers pertinent to the dental field (time span 2005–2010), searched with the topic keywords “Dental/Oral electrospinning” in the ISI Web of Knowledge database, while the articles became 39 in the time span between 2011 and 2015 (final year considered in the aforementioned reference). Searching the papers with the same methods we report an increased publishing activity in this field, with a total of 221 records between 2015 and 2019 (time of search November 2019), as reported in Table 2.

The most studied electrospun polymers in this field can be classified into natural or synthetic polymers; these can be also used in blends. Nanofibres prepared with synthetic polymers exhibit better mechanical properties than those based on natural ones. An interesting strategy to ameliorate nanofibres mechanical properties and bioactivity is to combine different synthetic polymers or natural polymers or even to mix natural with synthetic polymers [64]. Some examples of studied polymers are PCL (polycaprolactone) [51], PLA (polylactic acid) [65], PLGA poly(lactic-co-glycolic acid) [66], PTFE (polytetrafluorethylene) [67], alginate [68], hyaluronic acid (HA) [69] chitosan [70], silk fibroin [71], cotton cellulose [72]. These polymers have been also used in combination with mineral compounds such as hydroxyapatites or nano-hydroxyapatites, calcium phosphate, tricalcium phosphate, etc., as nanoscaled reinforcement and/or to improve the bioactivity. It has been shown that the performance of a simple polymer can be affected positively by the introduction of small amount (<1 wt.%) of nanoscale reinforcements [73]. The latest research is focused on functionalizing polymeric nanostructured electrospun membranes with antimicrobial and bone promoting agents. For the former action, the most commonly used agents are represented by amoxicillin [74], metronidazole [75], ciprofloxacin [76] (as antibiotics); polyvinylpyrrolidone [77], silver nanoparticles [70], zinc oxide [78]. For the latter property, BMPs [79], diphosphonates [80] and naringin [81] have been investigated.

The versatility offered by ELS make possible the preparation of bi- or multi-layered membranes, which can have different properties on the two sides of the membrane, corresponding to different tissue compartment in the surgical site of application [82]. Ideal membranes for guided bone regeneration should be biocompatible, space-making, permeable to fluids but acting as barriers for cells, slowly resorbable, bone-promoting and coupled with antimicrobial properties; expectantly not expensive. All the aforementioned properties can be reached starting from electrospun polymers or polymer mixtures, which result in nanostructured membranes with proper mechanical properties that can eventually be tuned with antimicrobial and bone-promoting compounds. Yang and co-workers successfully fabricated PLGA/HAp collagen/amoxicillin nanofibre membranes through coaxial electrospinning: in vitro analysis showed hydroxyapatite deposition on the membrane, a release of amoxicillin up to 40 h and no signs of fibroblasts on the opposite side of the membrane after 48 h of culture [83]. More recently, Lian and co-workers developed a bi-layered electrospun membrane with osteogenic and antibacterial properties based on a softer layer of PLGA/gelatin nanofibres incorporating dexamethasone-loaded mesoporous silica nanoparticles (DEX@MSNs), and a denser layer of PLGA nanofibres loaded with doxycycline hyclate (DCH). In vitro evaluation showed the effective antibacterial potency of the DCH/PLGA membrane together with an enhanced osteoinductive capacity for rat bone marrow stromal cells (BMSCs) [82]. Moreover, the group of He et al. verified the antimicrobial properties and bone formation induction of an electrospun composite membrane made of gelatin (Gln) and chitosan (CS) containing hydroxyapatite nanoparticles (nHAp) and (Pac-525)-loaded PLGA microspheres (AMP@PLGA-MS) [84]. Permeability was successfully tested for a PCL/PLGA electrospun membrane with the fluorescein isothiocyanate-bovine serum albumin (FITC-BSA; Sigma) used as a nutrient model [85]. Permeability through the membrane is important for the supply of nutrients and oxygen, and for the occurrence of the essential processes of bone regeneration, therefore one of the goals of the manufacturing of this PCL/PLGA [85] membrane for GBR was the addition of two hydrophilic additives to PLGA and PCL, respectively: Pluronic F127 (EG99PG65EG99; Mw 12,500; BASF, Parsippany, NJ, USA) and Tween 80 (polysorbate 80; Yakuri Pure Chemicals, Kyoto, Japan) in order to implement the resulting selective permeability and to enhance the pull-out strength. The authors stated that the two additives did not affect the mechanical strength of the obtained membrane significantly, as was reported elsewhere [86]. The state of the art of commercially available resorbable membranes is nowadays represented by the cross linked heterologous collagen-based (type I and type III, derived from swine) bilayer membrane Bio Gide^®^ (Geistlich Pharma AG, Wolhusen, Switzerland); whereas the non-degradable benchmark product is a titanium reinforced expanded polytetrafluoroethylene (ePTFE) membrane named Cytoplast^®^ (Osteogenics Biomedical, Lubbock, TX, USA). Hence, the paramount difference in terms of technology and fabrication processes is evident between the commercially available membranes and the state of the art of scientific research. At the time of writing this manuscript, the evidences reported in literature remains at the in vitro or in vivo (animal model, small sizes) level. Thus, someone might argue if the promising results reported by the basic research may be similarly good in a human application or even superior to the current outcomes. For example, Food and Drug Administration (FDA) regulates and authorizes implantable devices only after overcoming a long and expensive list of certifications and scientific evidence. Thus, researchers and companies have to make great efforts to transfer promising results of nanoscale engineered membrane in scientific research on humans thereafter to clinical practice.

## 6. Limitations of Electrospinning in GBR

Clinical use of membranes for GBR, as well as for all the other tissue engineering applications/strategies, requires biocompatibility and non-toxicity of the used compounds. Given the wide use of synthetic polymers to overcome mechanical limitations of natural substances, biocompatibility and non-toxicity are imperative also for cross-linkers and antimicrobial agents (e.g., silver nanoparticles).

Synthetic polymers may fulfil the structural and mechanical requirements in biomedical applications thanks to their tunable physical and chemical properties [87]. Thanks to their excellent biomechanical property, in vivo thermal stability, and biocompatible properties, most studies have focused on PCL nanofibres. Despite their structural strength, nanostructured PCL membranes are not rigid and cannot be molded. Hence ELS membranes are not suitable for non-self-sustaining bone defects. Space-making is one of the structural characteristics that, nowadays, cannot be reached by conventional electrospun membranes. Among several alternatives to ELS, centrifugal spinning could also produce nanofiber-based implantable devices [88,89]. In this procedure, the polymer jet is stretched by the centrifugal force instead of the voltage. From one side, centrifugal spinning could compensate some disadvantages of the ELS process (improving the mechanical properties of the structures and the spinning speed, to name some). On the other hand, ELS technique involves a set-up which is more simple that the one required for the centrifugal spinning.

**Figure 5:**
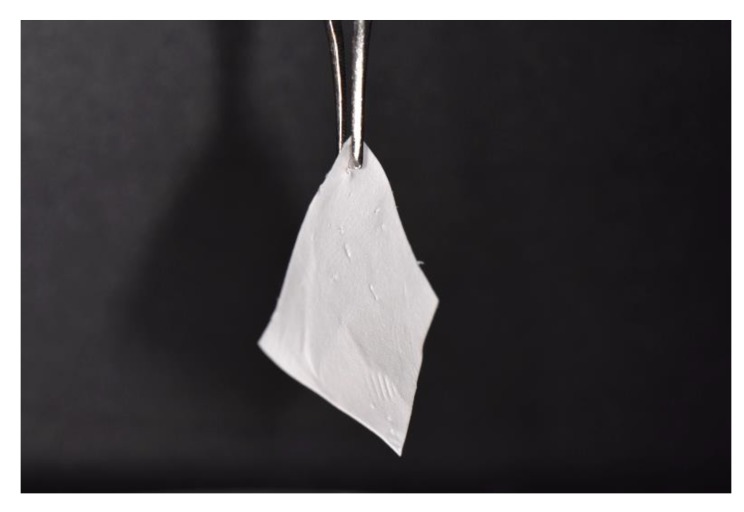
Nanofibre-based membrane obtained after 60 min of ELS with the following parameters: PCL 12% *w*/*v* in DCM/DMF 7:3, 17 kV potential, 27 G needle, 0.6 mL/h of flow rate. Nikon D3500, macro 105 Sigma tamron lens, Sigma ring flash. Membranes can be handled easily with surgical tweezers.

**Figure 6:**
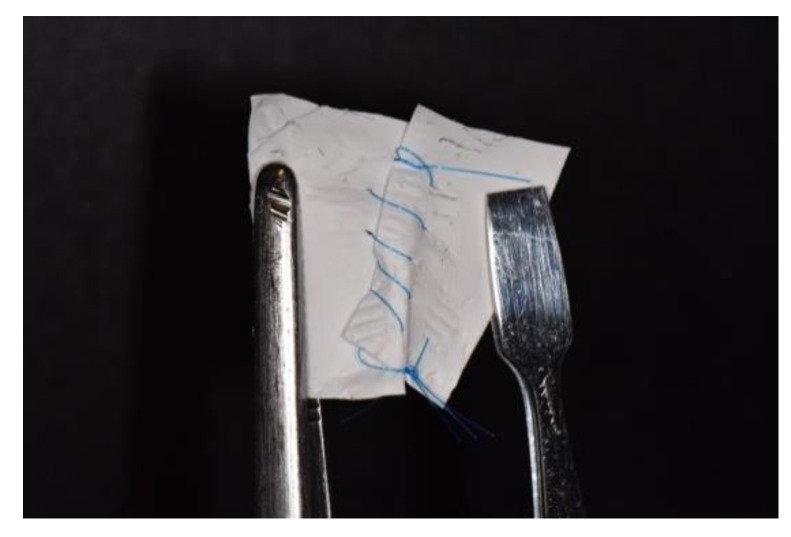
Nanofibre-based membrane obtained after 60 min of ELS with the following parameters: PCL 12% *w*/*v* in DCM/DMF 7:3, 17 kV potential, 27 G needle, 0.6 mL/h of flow rate. Nikon D3500, macro 105 Sigma tamron lens, Sigma ring flash. See the mechanical resistance during stretching the same membrane of Figure 5 after cut then sutured with 5/0 polypropylene.

According to its clinical use, a membrane should be handleable easily with forceps, maintain its mechanical properties after wetting in biological fluids and also it should be resistant to the shear forces of the surgical suture thread (suturable) (Figure 5 and Figure 6). Pull-out strength is an expression of mechanical properties of a biomaterial that in case of GBR setting is demanding. As an example, one main limitation of the PLGA/F127 membrane [86] was its relatively low suture pull-out strength and thus this membrane was not indicated for surgical applications in which sutures are needed [90]. To overcome this limitation, the authors published afterwards an augmented pull-out strength membrane, obtained adding PCL nanofibres to the solution. The PCL/PLGA membrane reached almost 8 N in the pull-out test, a result that is comparable with the gold standard Bio-Gide^®^, and to the benchmark threshold of 2 N (generally accepted for suturing during surgery) [91]. Moreover, when the PCL/PLGA membrane was soaked in saline solution, it showed a higher pull-out strength when compared to the wetted Bio-Gide^®^, which is more hydrophilic and, therefore, absorbent [85].

Membranes candidate for GBR should be carefully investigated in terms of permeability. This information is paramount to predict both the desired diffusion of nutrients and the undesired infiltration of host undesired cells (e.g., fibroblasts) through the structure. The albumin assay performed on the aforementioned membranes (PCL/PLGA) proved a similar, increase of permeability with time, when compared with controls (PCL/Tween 80 > Bio-Gide1 > PLGA/F127 > PCL/PLGA hybrid membranes) because of their hydrophilicity. Moreover the same paper provided an in vivo test of the bone regeneration capability. All the tested membranes in a calvaria bone defect rat model showed better results in bone regeneration when compared with the blank controls. Moreover, the PLGA/F127 > PCL/PLGA hybrid membranes showed a superior bone formation in terms of quantity and quality in respect to active controls. The common behavior found was a regeneration of bone starting from peripheric host bone allowed by the space making and selective permeability of the tested membranes, differently from the blank controls [85]. However, in vivo (with large size animal models) or human studies will clarify the role of dissolution time of the membranes in terms of mechanical performance that should be maintained at least for two months in the view of GBR applications.

In 2014, poly(butylene carbonate) (PBC) was tested as alternative of electrospun PCL for the production of membranes for GBR. In vitro and in vivo tests demonstrated similar behavior of the tested molecule to PCL; moreover a superior hydrophilicity was found for the PBC membranes. Interestingly, Young’s modulus of PBC and PCL membranes was 8.45 ± 0.93 and 9.91 ± 1.12 MPa, respectively; tensile strength was 3.53 ± 0.34 and 3.82 ± 0.36 MPa, respectively. This mechanical performance suggests a possible use in the field of GBR. Moreover, micro-CT images and histologic sections showed bone formation below both PCL and PBC membranes. Conversely, the blank control showed invasion of soft tissue in the calvaria defects in the murine model [92].

The choice of the right volatile solvent, mostly when natural polymer are used, is challenging. Reaching the proper evaporation of the solvents and obtaining nanofibres with homogeneous diameter and without defects represents a demanding task. To this end, a recently published paper found the best combination of solvents to face the dissolution of collagen and chitosan with hexafluoroisopropanol and formic acid respectively. The author successfully blended these two bioactive molecules demonstrating bone-promoting activity in in vivo tests [93].

Given the importance of pore dimension and density on cellular adhesion and differentiation, their modulation represents an important issue in membrane manufacturing. For bone tissues, the pore size of scaffolds is expected to fall in the range 100–500 μm [94,95]. Moreover, pore geometry influences cell morphology as well. In the conventional ELS set up, however, controlling pore dimension and distribution seems to be critical, according to the random deposition of the fibres on the collector. The use of structured collectors represents an easy way to generate greater pore size in electrospun scaffolds. Some authors report that this kind of collectors could positively affect the obtained structures essentially acting as a guided template that influences fiber collection during the deposition of polymers [96,97].

More recent attempts of developing an expanded 3D scaffold, that simulates more closely the extracellular matrix (ECM), have been performed using an aqueous sodium borohydride (NaBH_4_) solution. This treatment and the use of predesigned molds allowed a better control of the shape of the resulting scaffold. However, this method is not ideal as it requires the use of aqueous solutions, chemical reactions, and freeze-drying process that may interfere with polymers that are soluble in water [98]. Expansion of electrospun membrane has been recently studied with the use of the depressurization of subcritical CO_2_ fluid. The paper recently published by Keit and coworkers highlights how a traditional bidimensional nanofiber membrane may be transformed into a three-dimensional scaffold with desired thickness, gap distance, and porosity, to allow cell seeding and proliferation [99].

The technical limitations of traditional electrospinning strategies for bone-tissue engineering applications might be critical for the preparation of three-dimensional scaffolds [100,101]. There have been many attempts in the fabrication of 3D porous nanofibres scaffolds: for example, Song et al. described the preparation of a 3D porous scaffold via a layer-by-layer assembly of PCL nanofiber-based sheets prepared by electrospinning of PCL on a stainless steel 3D mesh [102]. By contrast, other techniques of casting or direct writing are already effective in the three-dimensional manufacturing of scaffold. For 3D scaffold production with ELS, a schematic illustration is provided (Figure 1 of the paper) by Yinxian and coworkers [103].

It can be stated that traditional ELS alone is an elective technology for membrane generation rather than for 3D scaffold but it can be combined with other scaffold preparation technologies for the production of 3D structures implemented with nanofibres.

Finally, the safety of the technical staff is mandatory during the utilization of an ELS device. The use of high voltage supply, together with the use of potentially toxic solvents may expose the personnel to chemical and physical risk [26].

A conventional laboratory set up of electrospinning device is quite cheap for membrane production, as well as the costs of the most commonly used polymers. However, the system shows a low production efficacy: the spinning of a few grams of polymer may take several hours. In a commercial setting this could be a critical issue that might be overcome only with an upgrade of the ELS setup (e.g., increasing the number of syringes) rather than the process itself.

## 7. Conclusions

Electrospinning represents a technique that has been investigated in depth in the field of tissue engineering. The capability of production of fibres of nanometric scale promotes the application of ELS for tissue regeneration purposes thanks to the high surface-to-volume ratio of these structures. In addition, the nanofibres architecture mimics the macromolecular network of the extracellular matrix. Its application in oral surgery for guided bone regeneration may be promising according to the chemical and biological, rather than mechanical, properties of electrospun membranes. Moreover, new approaches such as multifunctional multilayering and coupling with bone-promoting factors or antimicrobial agents, makes this technology very attractive. However, the current literature is growing only in terms of basic research with only a few in vivo studies in small animal models. Greater efforts should be made by researchers and companies to turn these results into clinical practice.

## Figures and Tables

**Table 1:** A list of the relevant parameters that may affect the final morphology of electrospun nanofibres is provided.

Solution Parameters	Process Parameters	Environmental Parameters
Viscosity	Voltage	Humidity
Concentration	Flow rate	Temperature
Conductivity	Shape of collector	Air flow
Dielectric constant	Needle gauge	-
Surface tension	Distance	-
Charge of jet	Angle	-
Solvent type	Motion	-
Polymer type	-	-
Polymer molecular weight	-	-
Polymer solubility	-	-
Boiling point	-	-

**Table 2:** Results of the bibliographic research in the ISI Web of Knowledge database between 2015 and 2019 (time of search November 2019).

	Electrospinning		Dental/Oral Electrospinning
Year	Topic Search	Title Search	Topic Search
2015	2477	567	29
2016	2617	547	33
2017	2911	612	49
2018	3137	593	47
2019	2949	503	63
